# Experimental Investigation of Environmental Factors Affecting Cable Bolt Corrosion in Simulated Underground Conditions

**DOI:** 10.3390/ma18153460

**Published:** 2025-07-23

**Authors:** Saisai Wu, Pengbo Cui, Chunshan Zheng, Krzysztof Skrzypkowski, Krzysztof Zagórski

**Affiliations:** 1 Joint National-Local Engineering Research Centre for Safe and Precise Coal Mining, Anhui University of Science and Technology, Huai’an 232001, China; chunshanzheng@aust.edu.cn; 2 Shanxi Key Laboratory of Geotechnical and Underground Space Engineering, School of Resources Engineering, Xi’an University of Architecture & Technology, Xi’an 710055, China; 3 Faculty of Civil Engineering and Resource Management, AGH University of Krakow, Mickiewicza 30 Av., 30-059 Kraków, Poland; 4 Faculty of Mechanical Engineering and Robotics, AGH University of Krakow, Mickiewicza 30 Av., 30-059 Kraków, Poland; zagkrzys@agh.edu.pl

**Keywords:** premature failures, cable bolts, mineralogical conditions, underground conditions

## Abstract

Corrosion-related failures have emerged as a critical driver of premature support bolt failures in underground mines, emphasizing the urgency of understanding the phenomenon with respect to enhancing safety in underground environments. This study investigated key factors influencing bolt degradation through extensive experimental evaluation of cable bolts in simulated underground bolt environments. Multi-stranded cable specimens were exposed to saturated clay, coal, mine water, and grout/cement environments. Water samples were collected weekly from critical packing sections and analyzed for pH, electrical conductivity, and dissolved oxygen. The mineralogy and atmospheric conditions were identified as principal corrosion factors, and clay-rich and coal matrices accelerated corrosion, linked to high ion mobility and oxygen diffusion. Secondary factors correlated context-dependently: pH was negatively associated with corrosion in mineral-packed environments, while conductivity was correlated with non-mineral matrices. Notably, multi-stranded cables exhibited higher localized galvanic corrosion in inter-strand zones, highlighting design vulnerabilities. This work provides pioneering evidence that geological conditions are primary drivers for corrosion-related failures, offering actionable guidance for corrosion mitigation strategies in mining infrastructure.

## 1. Introduction

Cable bolts, comprising steel wires wound around a central core, offer superior tensile strength of up to 60 tonnes and flexibility compared to rock bolts, making them indispensable in the application of an underground reinforcement system [1,2,3,4,5]. However, the complex geometry and material heterogeneity create localized stress concentrations that accelerate corrosion-related failures [6,7,8,9]. Corrosion-related failures result from the interaction between corrosive action and a stressed environment. For this form of corrosion to occur, a corrosive medium and a material susceptible to such corrosion are required. The failures caused by the stress level could be significantly lower than the material’s yield stress [10,11,12]. For pitting corrosion, the material’s strength may decrease drastically even with minimal degradation or crack development [13,14,15]. Recently, as underground mining operations have deepened, premature failures of rock and cable bolts induced by this ordinary corrosion have been observed more frequently [16,17,18,19]. This type of corrosion has thus emerged as a critical challenge in underground mining, compromising structural integrity and safety [20,21].

Laboratory investigations of bolt corrosion in simulated mine water environments have yielded critical insights into pH-dependent degradation mechanisms. A nine-month experimental study replicated US coal mine conditions using non-tensioned bolts submerged in solutions with controlled pH levels, systematically comparing corrosion rates, potential voltammetry, and open-circuit potentials [22,23]. Parallel research examined different rock bolt types in three saturated tanks maintaining a pH of 5.5 or 9, demonstrating environment-specific corrosion progression patterns [24,25,26]. Corrosion-related bolt failures have also been identified as a critical failure mechanism connecting bending loads to bolt degradation in Australian underground mining environments, with combined tension–flexural stresses accelerating fracture propagation through microcrack coalescence [27,28,29,30,31,32]. This degradation pathway has been quantitatively characterized using specialized testing apparatus capable of simulating complex loading regimes, enabling precise evaluation of crack initiation thresholds and corrosion–fatigue interactions [33,34,35]. The aforementioned studies have advanced our understanding of corrosion mechanisms, particularly galvanic corrosion and environmental factors like groundwater chemistry, and have also provided validated methodologies for assessing bolt service life under coupled mechanical–chemical degradation typical of deep mining operations. However, studies characterizing how site-specific environmental factors, such as groundwater chemistry, mineralogical interfaces, and grouting materials, interact to accelerate degradation still need to be conducted. Additionally, the effects of environmental factors like aerated water and ion concentration on mining contexts are underexplored. A lack of multifactorial and regional studies integrating real mining conditions as well as site-specific environmental interactions limits actionable insights for corrosion mitigation.

This study addressed the issues by refining experimental methodologies to evaluate corrosion drivers in underground bolting systems, with emphasis on cable bolts, and the role of clay, coal, mine water, and grout/cement environment in corrosion processes. A wider range of materials to accurately represent the numerous bolting situations in underground mines was designed. Cable bolt specimens were systematically incorporated to quantify alloy-specific degradation mechanisms, along with replication of in situ groundwater and mineralogical gradients. By monitoring pH, dissolved oxygen, ion concentrations, and electrochemical parameters, the corrosion drivers were classified. The findings contribute to the development of informed targeted corrosion mitigation strategies, enhancing the safety of underground support systems.

## 2. Materials and Methods

### 2.1. Testing Specimens

**Figure 1:**
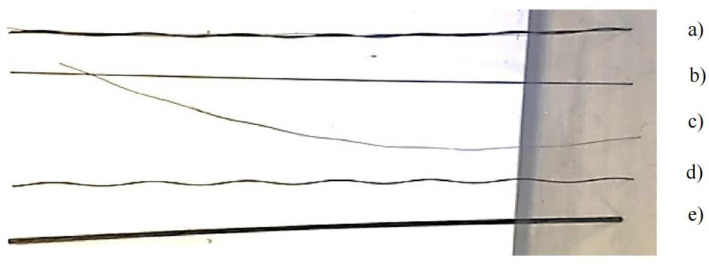
Tests of plain super strand specimens: (**a**) simplified cable bolt, (**b**) center wire, (**c**) outer strand, (**d**) inner strand, and (**e**) original cable bolt.

Plain super strand bolts were selected in the investigation for their widespread application in underground mining operations. The chemical composition of the specimen is detailed in Table 1 [36]. While the diameter of a standard plain super strand is 21.8 mm, a representative simplified model was developed to isolate and analyze corrosion interactions between strand components (Figure 1). The specimens of the cable bolts utilized in the corrosion testing were fabricated using SWPR19N steel (provided by the Jennmar, Sydney, Australia), which conforms to the Japanese Industrial Standard JIS G 3536 [37]. This modified design retained the central strand core but replaced the conventional multi-strand helical configuration with a single inner strand and a single outer strand, helically wound around the central axis. This simplified geometry eliminates confounding variables from complex multi-strand interactions, allowing targeted evaluation of localized electrochemical processes at strand contact points. This methods approach preserves the mechanical characteristics of full-scale cable bolts while enabling observation of microenvironments and interfacial corrosion behavior between individual strands.

### 2.2. Experimental Setups

To investigate corrosion-related failures in bolts within an underground mine, groundwater, clay, and coal samples were systematically collected. The groundwater samples, sourced from roof bolt seepage, exhibited notably elevated concentrations of sodium and sulfate, as detailed in Table 2. A discrepancy emerged in the corrosion risk assessment of this groundwater. According to the DIN 50929 standard [38], the water was categorized as posing a low risk to carbon steel. However, the WASM corrosive index predicted a corrosion rate exceeding 0.2 mm per year, suggesting the presence of a highly corrosion-prone environment [23]. X-ray diffraction analysis of the collected clay samples unveiled its composition: 96% kaolinite-group aluminum silicate hydroxide, 3% illite-group potassium manganese silicate hydrate, and 1% magnesium fluoride. Cation exchange capacity analysis further confirmed the clay’s alkaline nature. This characteristic enhances its ability to retain cations, potentially accelerating localized corrosion processes by facilitating the displacement of iron ions.

**Figure 2:**
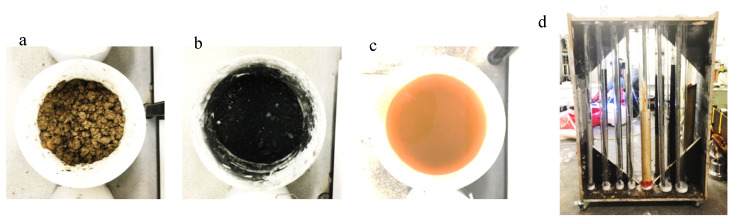
Test assemblies: (**a**) crushed clay, (**b**) crushed coal, (**c**) mine water, and (**d**) experimental frame.

To investigate the influences of mineralogical composition and atmospheric exposure on corrosion, this study developed an experimental framework with the defined variable classification system. Mineralogical packing types, including clay, coal, and grout–cement composites, were designated as independent variables. This design allowed for the isolation of material-specific corrosion mechanisms. Dependent parameters were carefully selected to characterize the corrosion process. Quantitative metrics were employed to track corrosion progression, including morphological classification and visual degradation pattern analysis. Additionally, groundwater chemistry profiles were monitored to understand the role of environmental factors, with parameters of pH fluctuations, electrical conductivity variations, dissolved oxygen, and ionic species concentrations being continuously measured. As outlined in Table 3, four different simulated corrosion conditions were designed through different packing media using the designed laboratory test methods (Figure 2).

As illustrated in Figure 2, the experimental setup featured a modular containment system fabricated from reinforced timber and steel fasteners. This system housed polyvinyl chloride tubes with a diameter of 200 mm. Each tube assembly was equipped with non-reactive polymer end caps sealed with silicone, ensuring fluidic isolation to prevent unwanted substance exchange. Adhesive membranes covered the atmospheric equilibrium ports, maintaining consistent gas-phase conditions during sampling periods. Paired irrigation ports connected to closed-end polymeric conduits regulated hydraulic pressure within the system. To facilitate localized fluid sampling, dedicated sampling ports were strategically positioned at the interfaces between coal and clay materials, as well as at bolt connection points. The entire apparatus was situated within a controlled environmental chamber, maintained at a constant temperature of 24 °C with a thermal variation of ±0.5 °C and a relative humidity of 95%. This experimental configuration integrated site-derived geological materials and interfacial configurations that mimic underground mining environments.

### 2.3. Testing Procedures

**Figure 3:**
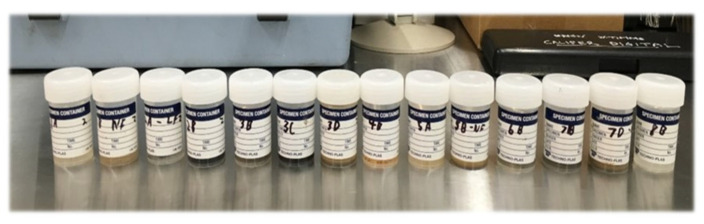
The labeled water sample.

**Figure 4:**
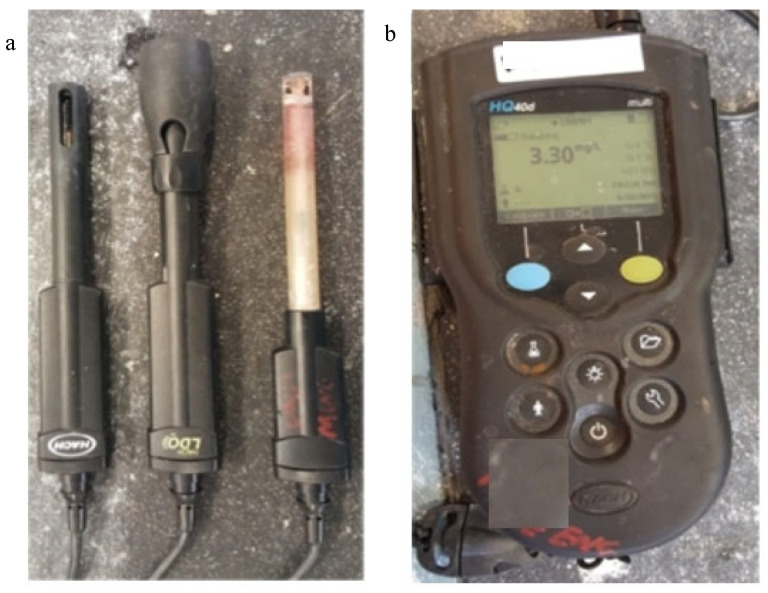
Water chemistry analysis devices: (**a**) EC, DO, and pH sensors; (**b**) water quality meter.

During the experimental period, water sampling was conducted weekly. From the designated ports, samples containing 40 mL of water were collected, labeled, and stored under controlled conditions to maintain sample integrity (Figure 3). Specialized extraction tools were utilized for sampling, including mechanical devices specifically designed for specimens encased in grout. To minimize sediment disturbance and maintain system equilibrium, after each sampling event, an equal volume of groundwater was sequentially injected from the bottom to the top of the system. Each collected sample was transported to the water analysis laboratory and analyzed for pH, electrical conductivity, and dissolved oxygen content using the LAQUA WQ-310-K water quality meter with EC, DO, and pH sensors (Figure 4). Sensors were calibrated weekly to ensure consistency across sampling periods. To ensure data consistency and reliability, sensors were recalibrated prior to each batch of measurements. At the conclusion of the tests, water samples underwent in-depth analysis through inductively coupled plasma optical emission spectroscopy (ICP-OES) and ion chromatography (IC).

Before analysis, all samples were filtered through 45 μm membranes to eliminate insoluble impurities, including mineral fragments and organic debris, that could interfere with the results. ICP-OES was employed to quantify elemental concentrations, and IC separated anions and cations by utilizing ion-exchange retention times. To establish correlations between hydrochemical data, ionic trends, and corrosion patterns, the obtained data were cross-checked with material logs and operational records. Trend analysis through linear fitting of the data was applied to identify data outliers caused by temporary flow obstructions, which were subsequently excluded from the final models. Following the completion of water sample analysis, the cable bolt specimens were carefully removed from the test assemblies for corrosion pattern examination. Surface deposits were non-destructively removed using high-pressure deionized water jets, ensuring that corrosion byproducts remained intact for subsequent analysis.

During the tests, electrochemical techniques and weight loss determinations were not used. This is because the complex multi-phase environment (involving groundwater, clay, and coal) introduces significant interference factors with respect to electrochemical measurements, such as unstable electrolyte conductivity and the presence of particulate matter that can disrupt electrode surfaces. This makes it challenging to obtain reliable and reproducible electrochemical data that accurately reflect the actual corrosion behavior. In terms of weight loss determinations, cable bolts have a complex surface morphology with helical structures, and corrosion products tend to adhere tightly to these irregular surfaces. Complete removal of corrosion products without damaging the base metal is difficult, which would lead to inaccuracies in weight loss calculations.

## 3. Results and Analysis

### 3.1. Water Chemistry Analysis

**Figure 5:**
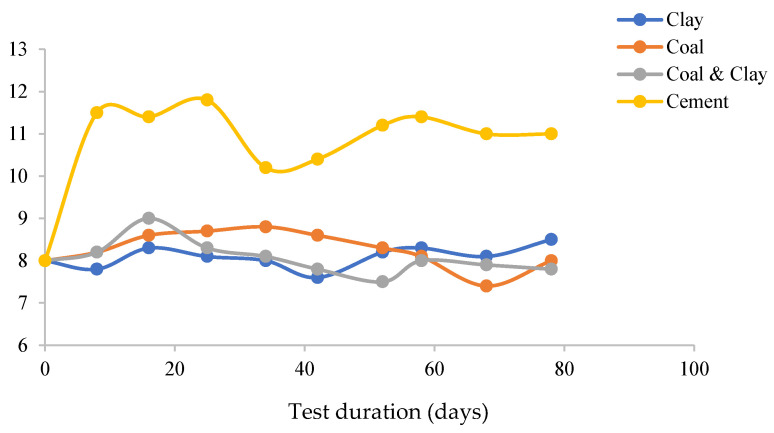
pH trends during the test.

Discrepancies in pH evolution were observed in the four experimental systems (Figure 5). In the clay–coal composite system, the pH value was stabilized at 8.1, primarily attributed to the buffering capacity of kaolinite-group minerals present in the clay. These minerals effectively regulated the hydrogen ion concentration, maintaining a relatively stable pH environment. The coal-dominated system exhibited a biphasic pH trend. Initially, oxidation of organic matter led to an alkaline shift, elevating the pH to 8.6. Subsequently, the breakdown of pyrite released sulfate ions, triggering acidification and causing the pH to decline to 7.4. This sequence of changes reflected the dynamic chemical reactions occurring within the coal matrix.

In the cement system, a consistently high bulk alkalinity near pH 11 was sustained through the diffusion of calcium ions during the cement hydration process. However, localized pH drops to 10.2 were detected, which were induced by chloride ions promoting electrochemical degradation and generating acidic byproducts. This observation underscored the delicate balance between the alkalinity derived from cement hydration and the acidic effects of electrochemical corrosion. The clay–coal mixture system moderated pH fluctuations by integrating the buffering ability of clay and the redox reactions of coal. This combination resulted in a more stable pH profile compared to the other systems. pH trends were in alignment with the observed ionic redistribution patterns. The increase in sulfate concentration in the coal system correlated with its acidification process, while the depletion of calcium ions in the cement system was consistent with alkalinity maintenance and localized pH changes. It is suggested that the interplay between mineralogy, chemical reactions, and ion dynamics is crucial in determining the pH behavior of different experimental systems.

These intricate pH dynamics not only reflect the inherent chemical properties of each system but also hold critical implications for understanding the corrosion behavior of cable bolts. The stability of pH in the clay–coal composite system, for instance, suggests that the buffering capacity of kaolinite-group minerals could mitigate extreme pH fluctuations that might accelerate or decelerate corrosion processes. In contrast, the biphasic trend in the coal-dominated system—from alkaline to acidic conditions—likely creates a shifting corrosive environment, where the initial alkaline phase might suppress corrosion while the subsequent acidification could enhance it, particularly in conjunction with the released sulfate ions.

**Figure 6:**
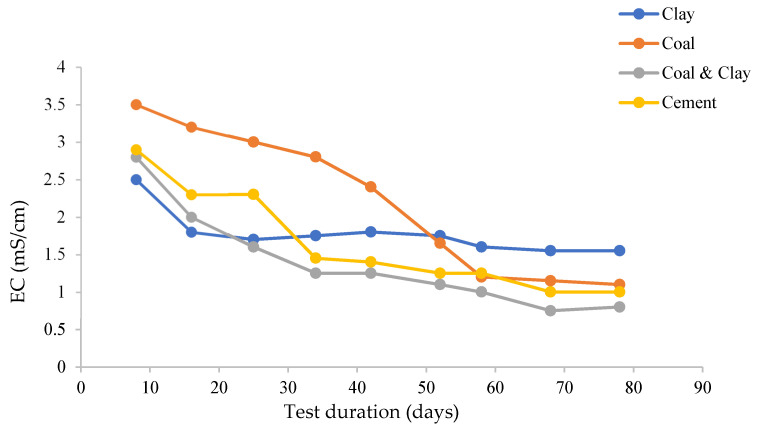
Electrical conductivity trends during the test.

The electrical conductivity trends in the four experimental systems were closely associated with material-specific ion transport mechanisms and chemical interactions (Figure 6). In the clay composite system, under near-neutral pH conditions (pH 8.2), the electrical conductivity stabilized at 1.8 mS/cm, with a standard deviation of 0.1. This stability was primarily due to the buffering effect of aluminosilicate minerals and the precipitation of gypsum, both of which suppressed ionic migration and limited the movement of charge carriers within the system. Conversely, the clay–coal composite system demonstrated a distinct conductivity pattern. Initially, conductivity dropped from 2.9 to 0.8 mS/cm over a period of 60 days, followed by a partial recovery to 1.2 mS/cm by the end of the test. This dynamic behavior was attributed to multiple factors: the accumulation of sulfate ions from pyrite oxidation, the cation exchange capacity of clay, the complexation of humic acids derived from coal, and the formation of an iron oxyhydroxide barrier. In underground mine settings, clay layers often interact with groundwater and surrounding organic-rich materials (including coal), creating conditions where humic acids from clay could coexist with those from coal. The presence of clay-associated humic acids would depend on factors such as the clay’s organic carbon content, its geological history of organic matter incorporation, and the redox and pH conditions of the local environment—all of which influence the formation and mobilization of humic substances. Thus, coal is a recognized source of humic acids. The synergistic mechanisms collectively influenced ion concentration and mobility, thereby altering electrical conductivity.

The cement system exhibited a conductivity trend similar to that of the coal system, characterized by an initial decline followed by stabilization. The release of calcium ions during cement hydration counteracted the acidity induced by chloride ions, maintaining a balance between bulk alkalinity and localized ion redistribution. This equilibrium process regulated the overall ionic concentration and movement, resulting in the observed conductivity pattern. The electrical conductivity patterns of the systems were consistent with the pH dynamics and ion behavior. The elevation of sulfate concentration coincided with the depletion of calcium ions. It was also observed that interfacial chemical processes, including mineral buffering and redox reactions, interacted with ionic equilibrium mechanisms.

The clay composite system’s stable conductivity creates an inert ionic environment which, paired with near-neutral pH, limits aggressive ion transport and reduces accelerated corrosion risks; in contrast, the clay–coal system’s fluctuating conductivity reflects chemical activity, with an initial drop temporarily slowing corrosion and a subsequent recovery signaling resurgent charge carriers that may intensify metal–surface reactions especially with dynamic pH, highlighting how conductivity shifts signal transitions in corrosion-promoting conditions. The cement system’s conductivity mirrors the coal system, balancing calcium ion release and chloride-induced ion redistribution, with stabilized conductivity suggesting ion equilibrium that influences the protective layer’s longevity, where low values may preserve passivation and spikes indicate barrier disruption, which are critical for assessing long-term corrosion risk.

**Figure 7:**
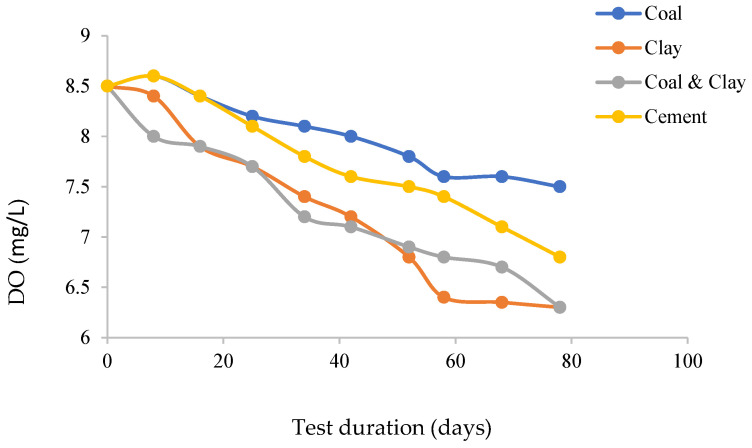
Dissolved oxygen trends during the test.

The dissolved oxygen trends across the four experimental systems demonstrated specific dynamics (Figure 7). In the coal composite system, dissolved oxygen levels remained relatively stable, fluctuating within the range of 7.5 to 8.5 mg/L. This stability was primarily attributed to the presence of organic constituents, such as humic acids, which suppressed oxygen depletion through radical scavenging mechanisms. These organic compounds effectively sequestered reactive oxygen species, maintaining a consistent oxygen concentration. The clay system exhibited a notable 35% decline in dissolved oxygen, decreasing from 8.5 mg/L to 6.5 mg/L. This reduction was driven by redox-active minerals, specifically iron oxides, present in the clay. These minerals accelerated oxidation reactions, consuming oxygen as a reactant and thereby lowering its concentration in the system.

Despite the high bulk alkalinity of the cement system, a gradual reduction in dissolved oxygen was observed. Chloride-induced acidity and microcracks generated during the hydration process created a paradoxical scenario. While high alkalinity might have been expected to inhibit oxygen-related reactions, the shift to acidic conditions—coupled with microcracks that facilitated localized ingress of atmospheric oxygen—instead promoted oxygen reduction reactions. The coal–clay composite system showed intermediate behavior, with the reductive properties of coal partially counteracting the oxygen-consuming redox cycles of clay. This interaction resulted in a dissolved oxygen trend that was less pronounced than in the pure clay system but more dynamic than in the coal-only system.

Dissolved oxygen trends, intertwined with each system’s chemical and ionic dynamics, critically shape cable bolt corrosion behavior: the coal composite system’s stable oxygen levels create a consistent oxidative environment with predictable, moderate corrosion rates; the clay system’s significant oxygen depletion shifts to reductive conditions, slowing aerobic corrosion but potentially promoting anaerobic pathways depending on factors like sulfate levels; the cement system’s gradual oxygen reduction, amid alkalinity and microcracks, reflects a dual effect where high bulk alkalinity tends to passivate the surface but microcrack-facilitated oxygen ingress and localized acidity drive localized corrosion; and the coal–clay composite system’s intermediate oxygen behavior, balancing coal’s reductive properties and clay’s oxygen-consuming reactions, results in a corrosion environment with more variable rates than the coal system but less abrupt shifts than the pure clay system. Collectively, these dynamics link chemical properties to corrosivity, offering insights into degradation intensity and mechanisms while complementing pH and conductivity observations.

### 3.2. Corrosion Patterns

Cable bolt corrosion manifested distinct patterns in the four experimental material environments, primarily governed by specific electrochemical mechanisms. Key features of corroded wires in different test conditions were summarized in Table 4. The experimental observations systematically revealed the relationships between material chemistry parameters, such as pH gradients and ion mobility, and interfacial dynamics, including oxygen accessibility and alloy heterogeneity. In coal-saturated conditions, localized pitting corrosion predominantly occurred at the upper segments of the cable bolts. The moderately acidic pH range of 6.8–7.2 and elevated electrical conductivity between 1.9 and 3.6 mS/cm facilitated chloride ion migration while restricting oxygen diffusion. These environmental conditions accelerated anodic dissolution processes, which aligned with the observed sulfate accumulation resulting from pyrite oxidation. The interplay of these factors created a conducive environment for localized corrosion attacks. In clay-saturated systems, galvanic corrosion was observed at the contact points of tri-wire assemblies. This phenomenon was attributed to the electrochemical potential differences among the steel alloys in contact. Despite the inherent corrosion inhibition properties of clay, the alkaline buffering capacity (pH 8.1–8.4) combined with its high cation exchange capacity led to the concentration of chloride ions at crevices. This localized chloride accumulation sustained the degradation process at the tri-wire interfaces.

The coal–clay composite environment exhibited a suppressed corrosion behavior, achieved through two primary mechanisms. The reductive properties of coal, along with the formation of carbonaceous surface coatings, effectively passivated the metal surfaces. Even with the presence of strontium enrichment due to clay adsorption, the stable pH of 8.1 and moderate conductivity of 1.5 mS/cm indicated balanced redox conditions, which inhibited aggressive corrosion reactions. In cement-grouted systems, oxygen-driven cathodic reactions were the dominant electrochemical process. Air intrusion during sampling events increased the availability of oxygen, thereby enhancing the production of hydroxyl ions and resulting in the release of ferrous ions within the range of 180–220 mg/L. Paradoxically, under these conditions, no visible signs of corrosion were observed on the cable bolts.

### 3.3. Ion Analysis Results

Ion analysis in the four experimental environments revealed distinct ionic redistribution patterns, each intricately linked to material-specific corrosion mechanisms (Table 5). The total dissolved solids (TDSs) exhibited significant variation: the coal-packed system had the second-highest TDSs due to organic matter dissolution, while the cement system showed the lowest TDSs, indicating baseline chemical stability. Notably, the coal–clay system recorded the highest TDSs, suggesting synergistic dissolution processes between the two materials. Elemental concentrations also differed markedly. Clay samples displayed extreme aluminum and iron levels. These elevated values were indicative of mineral hydrolysis and subsequent pitting corrosion. In contrast, the coal–clay mixtures suppressed the release of Al^3+^ and Fe^3+^/Fe^2+^ ions. This suppression was attributed to the reductive passivation effect of carbonaceous coatings derived from coal. The cement-grouted system maintained stable concentrations for most ions, with an unexpected depletion of iron. This depletion was likely due to iron re-precipitation as Fe(OH)_3_ under alkaline conditions characteristic of the cement environment.

In the highly alkaline cement system, the elevated pH induced iron precipitation, reducing its concentration relative to initial levels. Conversely, acidic hydrolysis in the clay system promoted the aggressive release of iron and aluminum ions. Electrochemical gradients systematically affected alkaline earth metals: calcium concentrations decreased by 12.3% in the cement system, while magnesium levels dropped by 8.7%, from 40.3 mg/L in coal to 3.6 mg/L in clay. Among anions, chloride mobility was most pronounced, increasing by 18.4% in the systems. Specific mineral-driven ion exchanges were also observed. Aluminum and iron ions emerged as key indicators of corrosion. Specimens with localized corrosion showed significantly higher Al^3+^ and Fe^3+^/Fe^2+^ concentrations compared to non-corroded samples, consistent with pitting corrosion mechanisms where the dissolution of Al and Fe dominates anodic reactions. System-specific electrochemical processes governed ionic redistribution through three primary pathways. In the cement system, pozzolanic reactions consumed silicon, while galvanic coupling led to a notable increase in manganese concentration. The reductive coatings in the coal system, confirmed by the presence of carbonaceous layers, suppressed metal release through surface passivation. Vertical ion gradients further validated these mechanisms, with a 28% decrease in calcium and a 22% increase in chloride accumulation, trends consistent with cathodic reaction dynamics. It was suggested that interactions with the environment had the most significant effect, followed by electrochemical potentials, with material interface effects being relatively less dominant. pH-mediated solubility controls, such as iron hydroxide precipitation, interacted synergistically with interfacial reactions.

## 4. Discussion

The experimental results revealed that corrosion mechanisms varied significantly in the different systems, primarily governed by the material-specific interactions among pH, electrical conductivity, and dissolved oxygen levels. In the coal–clay composite systems, generalized corrosion predominantly occurred at the upper segments of the specimens. Under stable dissolved oxygen conditions, humic acids derived from coal formed effective oxygen diffusion barriers. This mechanism maintained overall chemical stability with a consistent pH, despite localized corrosion processes taking place. Conversely, clay systems exhibited galvanic corrosion at the contact points of tri-wire assemblies. The electrochemical potential differences among steel alloys, coupled with the alkaline buffering capacity of clay, led to the concentration of chloride ions at crevices, thereby promoting localized degradation.

In cement systems, air intrusion during sampling events triggered oxygen-driven cathodic reactions. These reactions resulted in elevated ferrous ion release and increased electrical conductivity, reflecting enhanced electrochemical activity. Analysis of material interfaces indicated that they had a minimal impact on corrosion progression. The boundaries between coal and clay showed negligible variation in pH and electrical conductivity values, while the cement–water interfaces remained stable. These observations confirmed that bulk mineralogy played a more dominant role in determining corrosion behavior than interfacial interactions. The coal systems exhibited dual functionality in corrosion processes. On one hand, the oxidation of pyrite initiated pitting corrosion; on the other hand, organic coatings derived from coal suppressed the release of aluminum and iron ions. Vertical ionic gradient analysis revealed higher ion mobility in coal systems compared to clay systems, which correlated with the sulfate-driven conductivity spikes that were subsequently stabilized by gypsum precipitation.

It should be noted that results from scientific surface examination techniques such as optical microscopy, scanning electron microscopy (SEM), and X-ray photoelectron spectroscopy (XPS) could be extremely insightful, since these techniques would provide detailed information about the microstructure of the corrosion products, the morphology of the corroded surface, and the chemical composition of the corrosion layers, which would greatly enhance the depth and accuracy of our current corrosion analysis. The usefulness of detailed microscopic morphological and compositional information about corrosion products and surface changes is acknowledged. Such analyses would provide valuable supplementary insights into the corrosion mechanisms and degree of deterioration of the specimens, complementing the data on pH, dissolved oxygen, ion concentrations, and electrochemical parameters reported in this work. In future research, efforts will be made to address these constraints by performing a comprehensive surface characterization using the aforementioned techniques.

The study offers insights into cable bolt corrosion within simulated underground environments, though certain limitations should be noted that may affect the breadth and generalizability of its conclusions. The experimental design focuses on a specific set of simulated matrices, including saturated clay, coal, mine water, and grout/cement. This scope may not fully encompass the complexity of real-world underground settings, which often involve diverse geological formations like carbonate-rich rocks or saline layers, temperature fluctuations, and microbial communities such as sulfate-reducing bacteria that can influence biocorrosion processes. The investigation’s temporal framework, based on weekly water sampling, may not fully capture long-term corrosion dynamics. Seasonal shifts in groundwater chemistry, mineral dissolution rates, or sustained oxygen diffusion over extended periods could influence corrosion mechanisms in ways not fully reflected in the current data. While mineralogy and atmospheric conditions are identified as primary factors, the study did not fully explore interactions with other key variables. These include mechanical stress from bolt preload or microcracking caused by rock displacement, as well as electrochemical effects from stray currents common in mining operations, which may contribute to localized corrosion. Therefore, caution should be exercised, as laboratory simulations may not fully replicate the entire range of environmental stressors encountered in actual underground mining environments.

## 5. Conclusions

This study elucidates cable bolt corrosion mechanisms through extensive simulated underground experiments. Key findings demonstrate that mineralogical composition particularly in clay–coal systems and atmospheric exposure in aerated zones primarily govern corrosion initiation and progression. Clay and coal directly triggered corrosion onset, while atmospheric proximity and airflow rates modulated degradation kinetics. Secondary factors included pH—exhibiting significant corrosion correlation exclusively in mineralogy-packed systems—and electrical conductivity, whose corrosion relationship remained mineralogy-independent. Notably, galvanic degradation manifested uniquely in multi-strand cables, underscoring cable architecture’s critical role. Quantitative analyses reveal pH strongly correlates with corrosion rates in mineralogy-packed environments but not in grout. Elevated aluminum and iron ion concentrations indicated dissolution processes suppressed by passivation. Coal exhibited dual functionality: initiating pitting corrosion via pyrite oxidation while forming humic acid barriers that restrict ion migration, thereby mitigating corrosion. The obtained results resolve persistent ambiguities in underground infrastructure corrosion, providing actionable guidelines for material selection, cable design, and environmental management. The application of laboratory findings should be approached with caution, as laboratory simulations may not fully replicate the entire range of environmental stressors encountered in actual underground mining environments.

## Figures and Tables

**Table 1:** Chemical composition of cable bolt specimens [36].

Chemical Composition [wt.%]						
C	Si	Mn	Ni	Al	Cr	Fe
0.85	0.31	0.66	0.02	0.02	0.11	Balance

**Table 2:** Mine water chemistry.

Parameter	Unit	
pH	pH unit	7.8
Total dissolved solids	mg/L	398
Dissolved oxygen	mg/L	5.92
Sulfate	mg/L	80.5
Chloride	mg/L	7.4
Alkalinity	mg/L	355
Sodium	mg/L	182
Iron	mg/L	1.39

**Table 3:** Experimental designs.

Specimen ID	Packed Material	Specimens
1	Clay, Water	simplified cable bolt
2	Coal, Water	simplified cable bolt
3	Coal/Clay, Water	simplified cable bolt
4	Cement, Water	simplified cable bolt

**Table 4:** Key features of corroded wires in different test conditions.

Test Conditions	Clay	Coal and Clay	Coal	Cement
Appearance	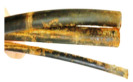	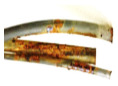	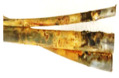	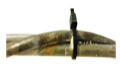
Removal of corrosion rust	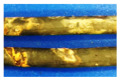	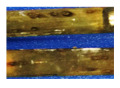	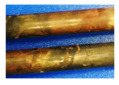	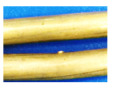
Dominant corrosion form	Galvanic corrosion (tri-wire contacts)	Suppressed corrosion of carbonaceous layers	Localized pitting	Oxygen-driven cathodic corrosion
Key features	Alkaline pH (8.1–8.4), high Cl^−^ trapping, alloy disparities.	Reductive coatings, Sr^2+^ enrichment, balanced pH (8.1) and conductivity.	Moderate pH (6.8–7.2), high Cl^−^ mobility, sulfate accumulation, low oxygen.	Alkaline bulk (pH~11), Fe^2+^ release, high mass loss.

**Table 5:** Ion analysis results with respect to different test conditions.

	Ca^2+^ (mg/L)	K^+^ (mg/L)	Mg^2+^ (mg/L)	Na^+^ (mg/L)	Si^+^ (mg/L)	S^2−^ (mg/L)	Al^3+^ (mg/L)	Mn^2+^ (mg/L)	Cu^2+^ (mg/L)	Sr^2+^ (mg/L)	Fe^3+^/Fe^2+^ (mg/L)	TDS (mg/L)
Coal	37.3	20.5	40.3	187.1	3.8	39.8	38.8	256.2	3.7	10.7	120.9	738.0
Cement	9.0	28.3	13.2	117.4	1.01	36.7	15.6	199.8	4.2	28.9	6.8	454.1
Clay	3.9	31.6	3.6	138.6	7.5	31.9	444.0	18.0	28.0	23.1	166.6	730.1
Coal/Clay	4.8	28.5	6.4	147.9	5.0	31.4	57.5	3.9	11.5	37.1	105.6	885.8

## Data Availability

The original contributions presented in this study are included in the article. Further inquiries can be directed to the corresponding authors.

## References

[1] Li C. (2010). Field Observations of Rock Bolts in High Stress Rock Masses. Rock Mech. Rock Eng..

[2] Wu S., Ma X., Zhang X., Chen J., Yao Y., Li D. (2024). Investigation into hydrogen induced fracture of cable bolts under deep stress corrosion coupling conditions. Tunn. Undergr. Space Technol..

[3] Wu S., Hao W., Yao Y., Li D. (2023). Investigation into durability degradation and fracture of cable bolts through laboratorial tests and hydrogeochemical modelling in underground conditions. Tunn. Undergr. Space Technol..

[4] Chen J.H., Saydam S., Hagan P.C. (2018). Numerical simulation of the pull-out behaviour of fully grouted cable bolts. Constr. Build. Mater..

[5] Li D., Li Y., Chen J., Masoumi H. (2021). An analytical model for axial performance of rock bolts under constant confining pressure based on continuously yielding criterion. Tunn. Undergr. Space Technol..

[6] Wu S., Zhang Z., Chen J., Yao Y., Li D. (2023). Characterisation of stress corrosion durability and time-dependent performance of cable bolts in underground mine environments. Eng. Fail. Anal..

[7] Gamboa E., Atrens A. (2003). Environmental influence on the stress corrosion cracking of rock bolts. Eng. Fail. Anal..

[8] Zou J.F., Sheng Y.M., Xia M.Y., Wang F. (2020). A novel numerical-iterative-approach for strain-softening surrounding rock incorporating rockbolts effectiveness and hydraulic-mechanical coupling based on Three-Dimensional Hoek-Brown strength criterion. Tunn. Undergr. Space Technol..

[9] Hadjigeorgiou J., Potvin Y. (2011). A critical assessment of dynamic rock reinforcement and support testing facilities. Rock Mech. Rock Eng..

[10] Wu S., Cao X., Zhu Y., Skrzypkowski K., Zagórski K. (2025). Examination of Stress Corrosion Cracking of Rock Bolts in Simulated Underground Environments. Materials.

[11] Peng Y., Timms W. (2020). Hydrogeochemical modelling of corrosive environment contributing to premature failure of anchor bolts in underground coal mines. J. Cent. South Univ..

[12] Woodtli J., Kieselbach R. (2000). Damage due to hydrogen embrittlement and stress corrosion cracking. Eng. Fail. Anal..

[13] Gamboa E., Atrens A. (2003). Stress corrosion cracking fracture mechanisms in rock bolts. J. Mater. Sci..

[14] Craig P., Serkan S., Hagan P., Hebblewhite B., Vandermaat D., Crosky A., Elias E. (2016). Investigations into the corrosive environments contributing to premature failure of Australian coal mine rock bolts. Int. J. Min. Sci. Technol..

[15] Nürnberger U. (2002). Corrosion induced failure mechanisms of prestressing steel. Mater. Corros..

[16] Parrot R. (1997). An Investigation into the Effects of Corrosion on the Mechanical Properties of Strata Bolts.

[17] Hadjigeorgiou J., Dorion J., Ghali E. (2008). Support system performance under different corrosion conditions. J. South. Afr. Inst. Min. Metall..

[18] Guo Q.F., Liu H.L., Xi X., Ma H.C., Pan J.L., Cai M.F. (2023). Experimental investigation of corrosion-induced degradation of rockbolt considering natural fracture and continuous load. J. Cent. South Univ..

[19] Vandermaat D., Saydam S., Hagan P.C., Crosky A.G. (2016). Laboratory-based coupon testing for the understanding of SCC in rockbolts. Min. Technol..

[20] Kang H., Wu Y., Gao F., Lin J., Jiang P. (2013). Fracture characteristics in rock bolts in underground coal mine roadways. Int. J. Rock Mech. Min. Sci..

[21] Yang Z., Xu S., Wang W., Li D. (2024). Experimental study on mechanical aging properties of self-swelling anchorage bolt under chemical corrosion. Int. J. Min. Reclam. Environ..

[22] Bylapudi G., Spearing A., Mondal K., Bhagwat A. (2019). Stress corrosion testing of roof bolt grade 60 steel in simulated underground coal mine atmosphere. Min. Technol..

[23] Hassell R., Villaescusa E., Thompson A.G., Kinsella B. (2004). Corrosion assessment of ground support systems. Ground Support in Mining and Underground Construction.

[24] Vandermaat D., Saydam S., Hagan P.C., Crosky A.G. (2017). Back-calculation of failure stress of rockbolts affected by Stress Corrosion Cracking in underground coal mines. Int. J. Rock Mech. Min. Sci..

[25] Niazi H., Eadie R., Chen W., Zhang H. (2021). High pH stress corrosion cracking initiation and crack evolution in buried steel pipelines: A review. Eng. Fail. Anal..

[26] Villalba E., Atrens A. (2009). Hydrogen embrittlement and rock bolt stress corrosion cracking. Eng. Fail. Anal..

[27] Chen H., Kimyon Ö., Ramandi H.L., Craig P., Gunawan C., Wu S., Manefield M., Crosky A., Saydam S. (2021). Microbiologically influenced stress corrosion cracking responsible for catastrophic failure of cable bolts. Eng. Fail. Anal..

[28] Crosky A., Smith B., Elias E., Chen H., Craig P., Hagan P., Saydam S., Hebblewhite B. Stress corrosion cracking failure of rockbolts in underground mines in Australia. Proceedings of the Seventh International Conference on Rockbolting and Rock Mechanics in Mining.

[29] Villaescusa E., Hassell R., Thompson A. Development of a corrosivity classification for cement grouted cable strand in underground hard-rock mining excavations. In Proceedings of 5th International Conference and Exhibition on Mass Mining.

[30] Craig P., Ramandi H.L., Chen H., Vandermaat D., Crosky A., Hagan P., Hebblewhite B., Saydam S. (2021). Stress corrosion cracking of rockbolts: An in-situ testing approach. Constr. Build. Mater..

[31] Wu S., Zhu M., Zhang Z., Yao Y., Li Y., Li D. (2025). Prediction and risk assessment of stress corrosion failures of prestressed anchors in underground mines. Int. J. Min. Reclam. Environ..

[32] Aziz N., Craig P., Nemcik J., Hai F. (2014). Rock bolt corrosion—An experimental study. Min. Technol..

[33] Toribio J., Ovejero E. (2005). Failure analysis of cold drawn prestressing steel wires subjected to stress corrosion cracking. Eng. Fail. Anal..

[34] Ramandi H.L., Chen H., Crosky A., Saydam S. (2018). Interactions of stress corrosion cracks in cold drawn pearlitic steel wires: An X-ray micro-computed tomography study. Corros. Sci..

[35] Sartola I., Aromaa J. (2003). Corrosion of Rock Bolts and the Effect of Corrosion Protection on the Axial Behaviour of Cable Bolts, SRM 2013—Technology Roadmap of Rock Mechanics.

[36] Wu S. (2018). Laboratory Based Investigation of Stress Corrosion Cracking of Cable Bolts. Ph.D. Thesis.

[37] (2022). Steel Wire Ropes.

[38] (2018). Corrosion of Metals—Corrosion Likelihood of Metallic Materials When Subject to Corrosion from the Outside—Part 1: General.

